# From Social Robotics to Ecological Cognitive Care: An Enaction-Based Umbrella Review on Neurocognitive Disorders

**DOI:** 10.3390/healthcare14010066

**Published:** 2025-12-26

**Authors:** Giuseppe Romeo, Daniela Conti, Santo F. Di Nuovo

**Affiliations:** 1 Department of Humanities, University of Catania, 95124 Catania, Italy; g.romeo.uni@gmail.com; 2 Department of Educational Sciences, University of Catania, 95124 Catania, Italy; s.dinuovo@unict.it

**Keywords:** socially assistive robotics, neurocognitive disorders, mild cognitive impairment, enaction, cognitive ecology

## Abstract

**Background:** As ageing populations grow, the prevalence of dementia and pre-dementia conditions is rising. Emerging approaches to neurorehabilitation emphasize not only performance-based outcomes but also holistic, experiential, and person-centred aspects of care. The extended mind thesis further highlights the potential role of external tools in supporting impaired cognitive functions. Within this ecological and experiential perspective, Social Assistive Robotics (SAR) may offer a multidimensional approach to address cognitive, emotional, and social needs in neurocognitive disorders. **Objective:** To synthesize current evidence on the effects of robotic interventions within an enactive framework integrating mind, body, environment, and technology. **Methods:** A systematic search was conducted in PubMed, Ovid Medline, Scopus, ScienceDirect, Springer, Wiley, IEEE Xplore, ACM Digital Library, and the Cochrane Library. Due to heterogeneity among included studies, an umbrella review was performed using vote-counting by direction of effect as a non-quantitative synthesis method. Methodological rigour followed JBI and Cochrane guidelines. **Results:** Sixteen reviews were included. The strongest and most consistent benefits emerged for affective outcomes, particularly emotional response and social interaction *p* = 0.007 (two-sided). Conversely, outcomes related to cognition, anxiety, agitation, depression, and quality of life showed mixed or non-significant effects, while neuropsychiatric symptoms demonstrated no benefit. **Conclusions:** Discrepancies across reviews seem driven by methodological limitations in primary studies, limiting interpretability. The strength of this umbrella review lies in identifying systematic gaps that can guide future research. With stronger evidence, integrating SAR into experiential neurorehabilitation may offer a promising avenue for holistic, ecologically grounded care that extends beyond traditional task-based performance. **Trial Registration:** PROSPERO CRD420251165419.

## 1. Background

In recent years, theories of the extended mind and distributed cognition have profoundly transformed how we understand cognitive processes and, consequently, how we approach cognitive rehabilitation. The Extended Mind thesis, introduced by Clark and Chalmers [1], argues that mental processes are not confined within the brain and body but can extend into the environment, including tools and technologies. In this view, everyday digital objects—such as smartphones, apps, and digital reminders—are not simply external aids but components of an extended cognitive system [2,3].

The enactive paradigm similarly proposes that cognition emerges from dynamic interactions among brain, body, and environment, shifting the focus of rehabilitation from restoring isolated functions to supporting new patterns of interaction and adaptation. Neurological damage, therefore, is not solely understood as a cerebral dysfunction but rather as a disruption in the dynamic interplay between the body and the surrounding world [4].

Taken together, these frameworks suggest a shift toward an ecological and distributed view of cognition, highlighting the helpfulness of technologically enriched environments in neurorehabilitation [4,5]. To formulate the rationale for the present study, these theoretical premises must be directly related to the clinical context of cognitive impairment and to the emerging role of Socially Assistive Robotics (SAR).

### 1.1. Cognitive Impairment: Clinical Context and Empirical Evidence for Social Robotics

According to the Diagnostic and Statistical Manual of Mental Disorders [6], dementia falls under the broader classification of Major Neurocognitive Disorder (Major NCD). This condition is defined as a marked decline of cognitive performance in one or more domains—such as attention, executive function, memory, language, perceptual-motor abilities, or social cognition—based on both the individual’s report and objective clinical or neuropsychological evidence. A key diagnostic distinction is that the cognitive impairment must be severe enough to compromise independence in everyday activities. This requirement differentiates Major NCD from Mild Neurocognitive Disorder (Mild NCD), where autonomy is generally preserved. Furthermore, Major NCD encompasses several etiologies, including Alzheimer’s disease (AD), vascular and frontotemporal pathology, Lewy body disease, and other medical conditions that impair brain function [6].

Within this nosological framework, clarifying the concept of Mild Cognitive Impairment (MCI) is essential. Although largely equivalent to Mild NCD [7], MCI is an additional clinical construct used to characterize individuals who exhibit cognitive decline greater than expected for age and education while maintaining autonomy. This condition occupies an intermediate position between typical ageing and early dementia and is associated with an increased—though not inevitable—risk of progression to Major NCD [8,9]. It is considered a condition of vulnerability, often prodromal to AD, but also a therapeutic window, as individuals retain cognitive and functional resources that can be targeted through intervention. This makes MCI a suitable context for evaluating innovative rehabilitation approaches informed by extended and enactive perspectives.

Recent empirical evidence supports this approach. Randomized studies have shown that personalized digital training programmes, such as the tablet-based TECH protocol, can preserve global cognition in individuals with MCI compared to controls [10]. Similarly, research on virtual reality interventions has demonstrated improvements in visuospatial memory and cognitive flexibility, highlighting the value of immersive and ecologically valid environments [11,12]. Cognitive assessment is likewise evolving: computerized tools have proven more sensitive than traditional tests in detecting early deficits in MCI, highlighting that cognitive extension applies not only to interventions but also to diagnosis [13]. Moreover, a recent meta-analysis shows that combining non-invasive brain stimulation with cognitive training can enhance rehabilitative effects in MCI [14] confirming that plasticity emerges from the dynamic interaction between the brain and external tools.

In this context, over the past decades, SAR has emerged as an innovative approach. Specifically, as recently indicated by Figliano et al. [15] social robots provide cognitive stimulation, socio-emotional support, and personalized interaction, fostering engagement in individuals with MCI. Evidence suggests that interaction with humanoid or animal-like robots can enhance attention, episodic memory, and executive function, while reducing loneliness and anxiety, thereby supporting sustained cognitive activity [15].

From the perspective of evaluation and diagnosis, standardized cognitive assessments are essential to measure the effectiveness of SAR interventions. The Montreal Cognitive Assessment (MoCA) is widely used to detect mild cognitive deficits and track clinical progress. Updated Italian norms provided by Conti S. et al. [16], accounting for age, education, and sex, enable reliable comparisons across studies and ensure objective measurement of cognitive outcomes. Additionally, studies by Di Nuovo et al. [17] and Rossi et al. [18] have explored the use of social robots as psychometric tools for cognitive assessment.

In summary, the theoretical models of extended and enactive cognition, combined with clinical evidence on neurocognitive disorders and technological tools such as SAR, collectively support a reconceptualization of neurorehabilitation as an interaction-oriented process. This framework directly motivates the research objective of the present study.

### 1.2. Research Objective and Rationale

Traditional neurorehabilitation, grounded in a “cognitive paradigm”, has typically approached cognition as brain-centred. As Martínez-Pernía [4] argues, this view narrows therapy to neural repair, neglecting the embodied, subjective, and environmental dimensions of recovery. To address this limitation, he proposes Experiential Neurorehabilitation (ENR) as a therapeutic model grounded in the enactive approach to cognition. ENR reframes care as the restoration of lived experience, emphasizing emotional, physical, and personal recovery rather than task performance alone.

In view of this, the integration of digital training, virtual reality, and SAR represents a promising multimodal strategy [19]. This approach simultaneously targets residual cognitive function, social engagement, and motivation, while intelligent and robotic systems allow continuous, individualized monitoring of progress [18]. Notably, SAR has been shown to mediate emotional engagement and motivation through user-robot personality matching [20]. Additionally, it can extend patients’ peri-personal space by integrating with their sensorimotor system and enhancing interaction with the environment [21].

Despite its potential, SAR faces challenges, including optimizing robot design for older adults, minimizing technological barriers, and validating interventions at a larger scale. For instance, qualitative studies indicate that older adults with MCI and their families often discontinue digital training due to perceived difficulties or decreased motivation [22], suggesting that technology adoption depends not only on cognitive factors but also on emotional and social ones. By ensuring that interventions are acceptable and tailored to individual needs, SAR can support cognitive function, enhance quality of life, and potentially contribute to slowing the progression toward dementia [23]. Thus, SARs—particularly those enhanced with adaptive AI—may align with ENR’s principles by supporting personalization and enabling socially grounded therapy within the clinician–robot–patient triad.

Given our exploratory aims, this study extends beyond evaluating intervention effectiveness and aligns with the broader purposes of umbrella reviews [24]. To structure this synthesis, we adopt a framework-based approach, using ENR as theoretical lens to guide data extraction, comparison, and interpretation. Framework synthesis enables researchers to identify areas of alignment or conceptual dissonance between the selected framework and existing data [25].

Thus, following recent developments in evidence synthesis, this study stands as a health-technology umbrella review [26] applying the ENR framework to evaluate the role of SAR for older adults with age-related neurocognitive disorders. Because the enactive framework is still novel and lacks standardized operational measures, a purely quantitative or confirmatory synthesis is not feasible. Accordingly, this review focuses on conceptual patterns, mechanisms, and theoretical alignment rather than on effect-size aggregation alone. We therefore examine how existing outcome measures relate to enactive principles narratively.

The following research questions (RQs) will be addressed:

**RQ1.**  *To what extent do SARs enhance cognitive outcomes across different stages of age-related neurocognitive disorders?*

**RQ2.**  *How do SARs influence affective–motivational factors that may mediate therapeutic effects across varying levels of impairment?*

**RQ3.**  *Which contextual (clinical, technical, sociocultural) and ethical factors facilitate or limit SAR integration within an enactive–experiential framework across the dementia spectrum?*

The goal is to outline the state of the art and identify promising directions for developing innovative neuro-rehabilitative approaches that truly harness the synergy between mind, body, environment, and technology.

## 2. Methods

This search was reported according to both the PRISMA guidelines [27] and the JBI guidelines for conducting umbrella reviews [24]. The study is registered in PROSPERO under the registration number CRD420251165419.

### 2.1. Deviation from the Registered Protocol

The population criterion was broadened from that specified in the original PROSPERO protocol to include dementia and other forms of severe cognitive impairment. This adjustment was made due to the scarcity of reviews focused exclusively on MCI and to capture the full spectrum of age-related neurocognitive disorders relevant to a comprehensive synthesis on SARs. The modification was introduced prior to data extraction and did not alter the overall aims of the review.

As we progressed through the screening phase, we further refined our inclusion criteria to clarify how we handled studies involving mixed populations and diverse interventions. These refinements also implemented prior to data extraction and did not alter the review’s overall scope or objectives.

### 2.2. Eligibility Criteria

The inclusion criteria were defined according to the Population, Intervention, Comparator, Outcome, and Study design (PICOS) mnemonic [28], as described below.

(i) Population: Systematic reviews whose primary studies included older adults diagnosed with MCI, dementia, or AD, according to recognized diagnostic criteria (e.g., DSM-5, Petersen’s MCI, NIA-AA, NINCDS-ADRDA). Reviews including mixed samples of healthy individuals or participants with other cognitive disabilities (e.g., older and younger adults or children with cognitive deficits) were eligible if results specific to cognitive decline were reported or if at least 70% of participants were aged 60 years or older. No restrictions were placed on gender, ethnicity, or geographical location.
(ii) Intervention: Interventions involved SAR, with or without pharmacological support. SAR was defined as robotic systems designed to provide social, motivational, or cognitive support through interaction rather than purely mechanical or motor assistance. Combined interventions (e.g., SAR + cognitive training, SAR + physical activity, or SAR + virtual reality) were included if the SAR component was clearly described.
(iii) Comparator: All types of comparators were eligible; thus, no exclusion criteria were applied based on comparator type.
(iv) Outcomes: Reviews were required to report at least one cognitive, affective, or functional outcome relevant to neurorehabilitation. Cognitive function was assessed using global or domain-specific tests such as the Mini-Mental State Examination (MMSE), Montreal Cognitive Assessment (MoCA), or other validated tools. Robot features such as acceptability, usability, and safety were considered when directly related to rehabilitative goals.
(v) Study Design: Eligible studies were systematic reviews with or without meta-analyses of empirical research evaluating SAR-based cognitive or neuro-rehabilitative interventions. Reviews based on Randomized Controlled Trials (RCTs) were prioritized. However, given the emerging phase of this technology, reviews including non-randomized designs were also eligible, consistent with the flexibility of the AMSTAR-2 appraisal tool [29].

Exclusion criteria were as follows: (i) studies addressing neurological or psychiatric conditions not classified as age-related neurocognitive disorders (e.g., post-stroke cognitive deterioration); (ii) interventions involving robotic exoskeletons, prosthetics, or industrial robots used solely for motor or physical rehabilitation; (iii) reviews addressing only the technical development or engineering features of SAR without human outcomes.

### 2.3. Search Strategy

To ensure comprehensiveness, a systematic search was performed on many discipline-specific databases encompassing medicine, psychology, and engineering/robotics. These were PubMed, Ovid Medline, Scopus, ScienceDirect, Springer, Wiley, IEEE Xplore, ACM digital library, and Cochrane Reviews within the Cochrane library. A date restriction was applied, including studies published from January 2015 to September 2025. Studies were collected in October 2025.

We developed the search strategy based on the eligibility criteria outlined above, using the PubMed format as a starting point to identify appropriate and controlled terms from the Medical Subject Headings (MeSH) vocabulary. After several pilot searches, we adopted a hybrid query combining MeSH descriptors and free-text keywords to capture both indexed and emerging concepts, as this approach yielded a higher number of relevant records. Additionally, since we observed that some databases index review articles under other publication types (e.g., as conference papers), we incorporated study design keywords such as “review” and “meta-analysis” directly into the search query for all databases to ensure comprehensive retrieval (Table 1).

For the Cochrane Library and Ovid Medline searches, both MeSH terms and free-text keywords were employed and entered into their respective search fields. The individual search components were then combined using the “OR” and “AND” Boolean operators as appropriate.

As detailed in Table 1, the PubMed query was adapted to match the syntax requirements of each database. In Springer, Wiley, and the ACM Digital Library, the adapted query was employed without further modification. For IEEE Xplore, the same query was used, but the truncation symbols (asterisks) were removed to comply with the platform’s syntax rules.

In Scopus, the search was restricted to the title, abstract, and keyword fields (“TITLE-ABS-KEY”) to improve specificity and reduce irrelevant results. Finally, the query was adjusted for ScienceDirect to align with that platform’s specific syntax requirements.

### 2.4. Selection Process

All identified records were uploaded into Zotero, where the two independent reviewers (GR and DC) screened each systematic review against the eligibility criteria yielding almost perfect inter-rater agreement (Cohen’s κ = 0.86). Discrepancies were resolved through discussion or, when necessary, consultation with a third reviewer (SFD).

During screening, we observed that several relevant studies were not retrieved by the primary PubMed query despite inclusion of appropriate MeSH and free-text terms. To minimize the risk of omission, we conducted a supplementary search using PubMed’s “Similar Articles” feature for each article included from PubMed to identify additional studies with related content. This approach aligns with recommended best practices for extending systematic review searches through related-article retrieval [30]. Any newly identified records were screened using the same eligibility criteria as records identified through structured database searches; they are reported under “Identification of studies via other methods” in the PRISMA flow diagram.

**Figure 1:**
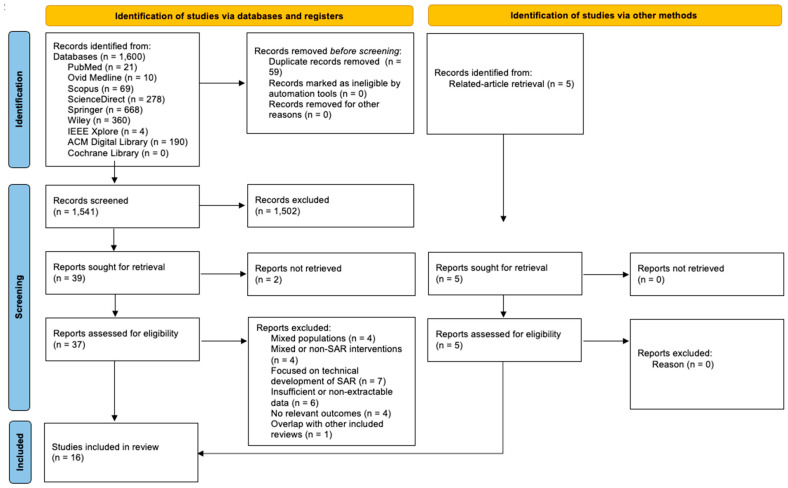
PRISMA flow diagram.

A total of 1600 records were identified through database searching, with an additional 5 records identified through related-article retrieval. After removing 59 duplicates and 1541 records before screening. Of these, 1502 were excluded at title/abstract stage. Thirty-nine full-text reports were sought for retrieval, and 37 were assessed for eligibility. Twenty-one reports were excluded for predefined reasons, and 16 systematic reviews were included in the final synthesis (Figure 1).

Lastly, to address duplication of evidence, overlap between primary studies included in the eligible reviews was evaluated and managed in accordance with the Methods for Overviews of Reviews (MOoR) framework [31].

### 2.5. Data Extraction

The data extraction was performed using JBI data extraction tool for Systematic Reviews and Research [24] and tailored in accordance with the research question and methodology. As for the studies selection, data extraction was performed in duplicate by two independent reviewers (GR and DC), with any disagreements resolved by consulting the third author (SFD). Only data relevant to the aims of the current umbrella review was extracted from included reviews.

### 2.6. Methodological Quality Assessment

Review quality was assessed using the AMSTAR 2 tool [29] which evaluates whether reviews had a clear research question and protocol, defined inclusion criteria, comprehensive search strategy, independent study selection and extraction, transparent reporting, and appropriate synthesis methods that account for bias. AMSTAR-2 deems six items as critical (i.e., protocol registration, search adequacy, bias assessment, meta-analytic methods, bias use in interpretation, and publication bias) and ten as non-critical. Thereby, confidence ratings were assessed as high (≤1 non-critical, 0 critical weaknesses), moderate (>1 non-critical, 0 critical), low (≥0 non-critical, 1 critical), or critically low (≥0 non-critical, >1 critical).

In addition to AMSTAR 2, risk of bias within included reviews was assessed using the ROBIS tool [32] which examines three domains of potential bias: study eligibility criteria, identification and selection of studies, data collection and critical appraisal, and synthesis and findings. Each domain was rated as low, high, or unclear risk of bias, and an overall judgement was made for each review.

### 2.7. Data Synthesis

Among the sixteen included reviews, eleven conducted meta-analyses reporting effect sizes but only six reported *p*-values explicitly. Due to this heterogeneity in outcome reporting, a vote-counting synthesis based on the direction of effect was performed to minimize subjectivity in conducting a non-quantitative synthesis.

This procedure served as a complement to our framework synthesis, which necessarily relied on the conventional clinical outcome metrics reported in the included reviews. To the best of our knowledge, no standardized operational measures of enactive or experiential constructs currently exist in the SAR literature; therefore, their enactive interpretation is developed conceptually in the Discussion section.

Following Cochrane guidance [33], for each outcome domain, the proportion of studies favouring the intervention was calculated (*p* = u/n), where u represents the number of effects favouring the intervention and n the total number of included effect estimates. A binomial sign test was applied to examine whether the proportion of favourable effects differed from the null hypothesis of no difference (0.5), and 95% confidence intervals for the proportions were computed using the Wilson method to quantify uncertainty. Consistent with Cochrane recommendations, statistical significance, effect size magnitude, and study quality were not used to determine the direction of effect.

Findings were organized into four domains: (i) cognitive outcomes (e.g., memory, executive function, global cognition); (ii) affective and motivational outcomes (e.g., engagement, mood, empathy, trust); (iii) functional and behavioural outcomes (e.g., independence, adherence, quality of life); and (iv) experiential and embodied dimensions of Human–Robot Interaction (HRI), interpreted through an enactive lens.

## 3. Results

### 3.1. Reviews Selection and Characteristics

The search strategy yielded a total of sixteen systematic reviews. The complete dataset of included and excluded records, along with their sources and exclusion justifications, is provided in Supplementary Materials (Tables S1 and S2).

The sixteen systematic reviews were published between 2017 and 2025 across distinct journals. Twelve appeared in medical journals, one in PLOS One, and two in engineering outlets. The latter adopted qualitative synthesis approaches [15,34,35], whereas most of the remaining reviews employed quantitative meta-analytic methods, including random-effects meta-analyses using Hedges’ g (e.g., [36,37,38,39]), fixed-effect models [40], and combined fixed- and random-effects approaches applying Cohen’s d depending on heterogeneity (e.g., [41,42,43]).

Table 2 summarizes the main characteristics of the included reviews. To avoid redundancy, participant conditions are not reported here, as all reviews included mixed samples of individuals with dementia (ranging from mild to severe) and MCI, primarily residing in nursing homes or long-term care facilities—except for one review focused exclusively on MCI [15]. Similarly, the comparator was consistently usual care across all studies. Geographic distribution is not tabulated given its heterogeneity, with studies spanning all continents except Africa. In all reviews, PARO (robotic seal) was consistently identified as the most studied social robot.

Finally, nearly all studies reported no conflicts of interest, except for the conference papers [34,35]. Only a few studies received funding, either from their respective universities [38,44] or from the National Research Foundation of Korea [36].

### 3.2. Quality of the Evidence

According to the overall AMSTAR-2 assessment, the methodological quality of the sixteen included reviews was rated as either “low” or “critically low”. Three reviews were rated as “low” because they all failed Item 7, a critical domain concerning the provision of a comprehensive list of excluded studies with justifications for exclusion. Detailed results of the AMSTAR-2 appraisal are presented in Supplementary Material (Table S3).

Using the ROBIS tool, only three reviews were judged to have a low risk of bias [15,39,43]. Most of the high-risk ratings were attributable to the absence of sensitivity analyses or assessments of publication bias, which could have strengthened the robustness of their conclusions. The detailed results of the ROBIS assessments are provided in Supplementary Material (Table S4).

As stated in Section 2.7, no review was excluded from vote counting in accordance with Cochrane recommendations. Nevertheless, methodological quality was considered during result interpretation and each review’s findings were prioritized according to their certainty (Section 4).

### 3.3. Primary Studies Overlap Across Included Reviews

Overlap among reviews was assessed using the Corrected Covered Area (CCA), which compares the number of repeated primary studies to the total number of possible citations [45]. The CCA was 6.67%, suggesting a moderate overlap. The complete citation matrix is available in the Supplementary Material (Table S5).

Although current guidelines recommend prioritizing only the most comprehensive and high-quality reviews [45], all reviews were ultimately included. This decision was made because, despite shared evidence bases, the reviews reported slightly divergent findings. In line with the umbrella review focus on identifying consistencies, discrepancies, and gaps in the evidence [26], we decided to offer a comprehensive overview of the literature useful for understanding consistency and variability across different reviews. This approach is consistent with the MOoR framework [31].

### 3.4. Results of Vote Counting and Narrative Synthesis

The findings of vote-counting synthesis based on the direction of effect are Summarized in Table 3.

#### 3.4.1. Cognitive Outcomes

Across the included reviews, cognitive function was predominantly evaluated in terms of global cognition using standardized instruments such as the MMSE or MoCA. Only two reviews [15,34] provided more detailed analyses of individual cognitive domains, including memory, executive function, and verbal communication. Overall, the evidence indicated a tendency toward no significant effect of SARs on cognitive performance. However, three reviews reported improvements in global cognition—two based on qualitative syntheses [15,34] and one from a quantitative meta-analysis [36]. Thus, the proportion of reviews reporting cognitive benefits was 0.27 (95% CI 0.09–0.56), and the two-sided sign test indicated that this distribution was not statistically different from chance (*p* = 0.22), suggesting that current evidence is inconclusive regarding the cognitive benefits of SARs.

#### 3.4.2. Affective and Motivational Outcomes

Evidence from the included reviews suggested that SARs may help reduce loneliness and enhance social interaction, mood, and positive affect. Quantitative estimates of effect size were available in only two meta-analyses [37,40], whereas most evidence for these outcomes was derived from qualitative or mixed-method syntheses [15,34,35,40,44,46,47]. All reviews reported improvements in social engagement and positive affect, and the sign test confirmed a statistically significant direction of benefit for these outcomes (*p* = 0.007).

In contrast, results for anxiety indicated modest improvements (0.60; 95% CI 0.31–0.83) that did not reach statistical significance (*p* = 0.75), while evidence for depression was mixed: the proportion favouring improvement was 0.45 (95% CI 0.21–0.72) and did not differ from mere chance (*p* = 1.00). Also, evidence for neuropsychiatric symptoms consistently showed no benefit (u = 0; *p* = 0.016). Most notably, one qualitative review reported increased hallucinations [34].

Finally, both engagement and acceptability outcomes consistently showed a positive direction of effect, but the magnitude of improvement was not quantified in most studies [15,34,35,44,46,47]. Even so, personalization and preferences for robot appearance and functionalities still require further investigation [35].

#### 3.4.3. Functional and Behavioural Outcomes

A moderate proportion of reviews favoured reductions in agitation (0.64; 95% CI 0.35–0.85), but this trend was not statistically significant (*p* = 0.55). Evidence for behavioural outcomes such as sleep and physical activity was inconsistent across studies [42,43,44]. Only a few reviews reported on other indicators, including adherence to treatment [34], improvements in activities of daily living [35], and reduced medication use, as robots appeared to distract patients from pain and anxiety [37,44,47]. Given the limited and heterogeneous evidence, vote counting was not performed for these outcomes; reviews recommend further examination on physiological outcomes [43,48]. Lastly, quality of life tended toward no benefit, with a proportion of 0.18 (95% CI 0.05–0.47) in favour, although this result did not attain statistical significance (*p* = 0.065).

#### 3.4.4. Experiential Rehabilitative HRI

As expected, none of the included reviews explicitly assessed outcomes using enaction-based or experiential metrics. Nevertheless, several findings can be interpreted through an enactive and ecological lens. Evidence of improvements in social interactions and engagement, suggests that therapeutic benefit may arise from embodied interactional processes rather than from information-based stimulation alone. This perspective is elaborated in the Discussion section (see Section 4.3).

## 4. Discussion

Findings were organized across two analytical levels: a first-level synthesis of domain-specific empirical outcomes (see RQ1, 2), and a second-level conceptual integration aimed at capturing their enactive and experiential adherence (see RQ3).

### 4.1. RQ1: To What Extent Do SARs Enhance Cognitive Outcomes Across Different Stages of Age-Related Neurocognitive Disorders?

Statistical evidence was not strong enough to reject the null hypothesis, indicating no consistent effect of SARs on cognitive outcomes. The uncertainty in direction of effect likely reflects substantial heterogeneity across primary studies and reviews, including mixed clinical populations, variation in cognitive impairment severity, and the use of diverse and insufficiently standardized cognitive training protocols. In line with the methodological expectations of umbrella reviews, we will examine and articulate the underlying reasons for these discrepant findings [24].

According to one of the three reviews with the lowest assessed risk of bias, cognitive exercises on memory were often administered without differentiating between mild and severe dementia, or between amnestic, non-amnestic, and multidomain MCI subtypes, making effects difficult to detect [15]. Moreover, executive functions were rarely targeted directly (unless considered indirectly in social trainings like playing bingo), and structured intervention protocols were often poorly described [15].

The largely non-significant results may also reflect the progressive and degenerative nature of dementia [41], where cognitive decline is unlikely to respond to physical, behavioural, or social stimulation alone [42,43].

The single meta-analysis reporting significant improvement [36] attributed its effect to robots’ two-way dialogue capabilities; however, this finding was not replicated in other reviews, which have attributed the insignificance of SARs effect to methodological limitations in primary studies. Notably, those studies reporting improvements in brain activity or delay of cognitive impairment still involved small samples and brief intervention periods [44], whereas cognitive change cannot be reliably assessed over short or mid-term timelines [42]. Additionally, subgroup analyses suggest that participants with MCI may benefit more than those with moderate-to-severe dementia [38], consistent with findings in autism, where SARs are more effective in mild and moderate cases than in severe ones [49]. This underscores the need for distinction by cognitive level, and the use of scales appropriate to participants’ cognitive functioning [47].

Taken together, these findings suggest that clinical severity may moderate the effectiveness of SAR interventions, with more severe conditions potentially diminishing responsiveness. Intervention dosage is another likely moderator. However, no review examined moderating factors systematically. Evidence from broader computerized cognitive training indicates that the impact of cognitive interventions can depend on both baseline impairment and training intensity. For instance, cognitive training may yield greater improvements in global cognition for individuals with AD and other dementias than for those with MCI [50]. Optimal training dosage also appears to vary by age: for adults under 60, the most effective regimen was 25 to <30 min per day, six days a week, while for those aged 60 or older, it was 50 to <55 min per day, six days a week [51]. Nonetheless, whether these findings hold for robotic interventions remains to be fully examined.

Overall, evidence for SAR-based cognitive improvement remains statistically non-significant and inconclusive due to heterogeneity in populations, intervention protocols, short study durations, and limited follow-up.

### 4.2. RQ2: How Do SARs Influence Affective–Motivational Factors That May Mediate Therapeutic Effects Across Varying Levels of Impairment?

Across reviews, the direction of effect on affective outcomes was generally positive, although the strength of evidence varied substantially by outcome. The most consistent findings emerged for emotional response and social engagement, where all included reviews reported improvements. Improvements extended beyond single HRIs, promoting interaction among residents and between residents and staff by enhancing social and communication skills [15,34]. These effects have been attributed to increased social stimulation and behavioural engagement, which may reduce physiological arousal and promote emotional regulation [42], in ways comparable to real pet therapy [41].

However, despite clear directional agreement, measurement inconsistency remains a major limitation, as primary studies did not use standardized measures for emotional state, affect, and social interaction [47]. This limits comparability across studies and may obscure subgroup effects.

One area where consensus was not reached concerns delivery format. Some reviews suggest stronger effects in group-based activities [44], while others highlight benefits of 1:1 interaction [37,47]. Importantly, this variation did not materially affect the overall directional trend in affective outcomes, which remained strongly positive.

Motivational engagement was also generally described as improved, although this finding was derived from only a few studies and was therefore not included in the vote-counting analysis. Patients appreciated the CompanionAble initiative and active stimulation through cognitive training and activity reminders (e.g., eating, drinking, taking medications, going out, or making video calls). Compared with other technologies such as tablets or computers, SARs offer an advantage by actively mediating therapeutic adherence through their autonomy [46].

In contrast, quality of life displayed a clear trend toward no benefit, with the sign test approaching closely but not reaching significance. This uncertainty likely reflects the multidimensional nature of quality of life, which encompasses physical health, mental state, degree of independence, social relationships, personal beliefs, and the environment [40]. In fact, reviews highlight that improvements tend to emerge when SAR interactions support autonomy or functional independence [35,48].

Finally, depression showed no reliable directional effect. Subgroup analyses suggest that group-based programmes may be beneficial [38,39], and intervention duration may also be a moderator, with 60–120 min weekly exposure [48], and ≥12-week interventions showing stronger effects in some analyses [39,43]. However, the optimal format, dose, frequency, and duration remain unclear. On average, most primary studies have used a low dose (15 to 30 min per session; rarely up to 60 min), high frequency (up to three times per week), and a mid-duration (8 to 15 weeks; in very few cases 30 weeks).

Overall, affective outcomes show the strongest and most consistent benefits in domains directly related to emotional expression and social interaction, whereas evidence for anxiety, agitation, depression, and quality of life remains mixed or not statistically significant.

### 4.3. RQ3: Which Contextual (Clinical, Technical, Sociocultural) and Ethical Factors Facilitate or Limit SAR Integration Within an Enactive–Experiential Framework Across the Dementia Spectrum?

Although SARs have not consistently demonstrated improvements in cognitive function or overall quality of life, the included reviews suggest that they can still contribute meaningfully to dementia care by supporting emotional well-being. From an enactive perspective, the lived experience of older adults with NCDs is shaped by behavioural and psychological symptoms of dementia [37,40,41,48], and by relational disruptions associated with loneliness [39]. People living with these conditions often believe that family members or others avoid spending time with them because of their dementia identity [46].

Yet, loss of social connection is associated with accelerated cognitive decline [46]. Accordingly, SARs could serve as a form of social prescription [36], with their therapeutic value arising from experiential, relational, and embodied interactions that, as noted, extend beyond the HRI itself.

Clinically, SARs offer non-pharmacological interventions [39,41], that can reduce caregiver workload while continuously monitoring patients around the clock [36]. They can be delivered flexibly in either group or individual formats, depending on clinical goals. For example, group sessions may enhance engagement and foster a social atmosphere, whereas 1:1 sessions may help concentrate on the individual’s specific needs such as building self-confidence or communication skills [37]. This adaptability supports a form of experiential care, where therapeutic benefit emerges through interactive and person-centred human–robot coupling.

However, several challenges remain. Sociocultural acceptability varies widely, shaped by gender, cultural background, and country differences [39]. Notably, reported preferences vary across regional contexts [41]. These contextual differences highlight the importance of tailoring SAR interventions to local norms, trust dynamics, and aspects of personal identity [52].

From a technical perspective, limitations include unreliable speech recognition in humanoid robots, dependence of telepresence robots on stable internet connectivity [48], and the significant costs associated with equipment, training, and ongoing maintenance [37]. Robots also lack the emotional depth, empathy, and complex decision-making abilities of human caregivers, which may limit their usefulness in neuropsychiatric care [43]. These challenges are further compounded by the digital literacy demands placed on both caregivers and patients [34,35]. Consequently, simpler and more intuitive interface designs are needed for patients [46] and staff alike [48], especially since professionals are also susceptible to mis-calibrated trust in AI when systems are not readily understandable [53].

Ethical considerations also influence both feasibility and real-world integration. Key concerns include the risk of infantilizing older adults, potential privacy intrusions from teleoperated systems, reduced human contact when robots replace rather than complement caregivers [44,48,54], and fears of workforce displacement [36]. Cost remains another significant barrier to equitable access [34,48]. Also, from the patient perspective, issues such as inappropriate emotional attachment and social neglect arise when users misinterpret the actual capabilities of the machines they interact with. Because humans naturally display empathy during HRIs, disruptions caused by technical failures, model obsolescence, or upgrades to SARs can become distressing or even upsetting for users [34].

Across these contextual and ethical dimensions, the literature consistently emphasizes the need for collaborative, interdisciplinary development. Enhancing acceptability, feasibility, and experiential quality requires ongoing partnerships among clinicians, families, engineers, and social and behavioural scientists at all stages of research and implementation [48]. Such collaboration is essential for embedding SARs within an enactive and ecologically grounded approach to dementia care, where meaningful therapeutic activity arises from the relational dynamics between patients, caregivers, and technology.

### 4.4. Limitations

This umbrella review contributed to identify both consistent and divergent findings, as well as systematic gaps in the existing literature. However, these results reflect several limitations.

First, we acknowledge the limitations of the type of analysis used for the quantitative part of the review. Vote-counting is used for study syntheses when the original publications provide limited information on the existence and direction of significant differences, preventing more sophisticated analyses due to the insufficient quantity and quality of the available data [55]. The vote-counting approach assigns equal weight to all studies regardless of sample size, methodological rigour, or effect magnitude. This equal weighting represents a methodological constraint, as it may mask the contribution of higher-quality evidence and lead to overly simplified conclusions. Moreover, because the included reviews exhibited a moderate degree of overlap, vote-counting does not yield statistically independent evidence. This constrains any inferential interpretation of sign-test results and limits conclusions to descriptive convergence only.

Second, the included reviews often reported inconsistent results despite drawing on largely overlapping sets of primary studies. The absence of systematic moderator analyses for each outcome (with the exception of Yen et al. [39]) further limits the ability to interpret these discrepancies. Although the overlap is moderate, the assumption of independence is violated.

Third, the primary literature displays several methodological weaknesses, particularly in relation to allocation concealment and assessor blinding [42,44]. Surely, the coupling between low quality evidence overlaps and vote-counting reduces the robustness and generalizability of the conclusions.

Additional limitations concern cognitive outcomes assessment specifically, including heterogeneous populations, variability in cognitive impairment severity, non-standardized intervention protocols, insufficient targeting of specific cognitive domains, and inadequately described procedures.

Finally, given that no enactive measures explicitly exist, the discussion could extract enactive meaning from conventional outcomes.

## 5. Conclusions

Drawing on Martínez-Pernía’s [4] proposal for Experiential Neurorehabilitation (ENR), this umbrella review examined the role of SARs in the care of individuals with neurocognitive disorders. By adopting an ecological perspective—one that emphasizes the dynamic interaction between mind, body, environment, and technology—we sought to situate current evidence on SARs within a broader rehabilitative framework rather than focusing solely on cognitive task performance.

SARs possess considerable potential for adaptable and personalized rehabilitation, and many studies already address variables aligned with the emotional, physical, and experiential dimensions emphasized in ENR. However, because of substantial heterogeneity across primary studies and reviews, we used a non-quantitative approach based on vote-counting by direction of effect. With the exception of affective outcomes—specifically emotional response and social engagement, which demonstrated consistent evidence of benefit—and neuropsychiatric symptoms, which showed a consistent lack of benefit, most outcomes (cognition, anxiety, depression, and quality of life) remained inconclusive, with sign-test results indicating no statistically reliable direction of effect.

The limitations identified in Section 4.4 reduce the value of the empirical review as a support for theoretical discussion. However, as noted by methodologists of meta-analytic syntheses (e.g., [56,57]) in certain innovative fields of study, the review must necessarily take into account the provisional nature of the results obtained and the shortcomings due to methodologies that are not yet refined. In these cases, the review of studies is a summary representation of the “state of the art” and a stimulus to conduct research with more appropriate methodologies that allow for stronger and more consolidated conclusions.

Consequently, several recommendations for future research emerge. First, improvements in RCT quality are needed. For cognitive outcomes, future studies should use clearly defined populations, standardized intervention protocols (e.g., distinguishing facilitated from non-facilitated formats; individual versus group sessions), explicit domain targeting (e.g., differentiating executive function from memory), and appropriate neuropsychological instruments, ideally complemented by neuroimaging measures. Standardized psychological assessment tools are also needed for affective outcomes.

Small sample sizes remain a constraint across studies. Moreover, evidence remains insufficient to determine whether robot type meaningfully influences outcomes. Previous research has shown that differences in robot morphology, degree of anthropomorphism, and interaction capabilities may lead to heterogeneous results (e.g., [58,59]). The reviews included in this umbrella analysis offer conflicting interpretations on this point, underscoring the need for more systematic comparative research. Additional research should also examine SAR effects on physiological parameters (stress, sleep, or physical activity) and medication use, which remain underexplored. Also, longer intervention durations are required to meaningfully evaluate outcomes, as cognition, depression, and quality of life, which are unlikely to show measurable change in short-term studies.

In conclusion, future investigations should prioritize examining moderator factors such as clinical subtypes, session duration and frequency, cognitive load intensity, the level of facilitation, format (individual vs. group), and the cognitive domain targeted. In addition, although SAR interventions conceptually align with enactive and ecological framework of ENR, substantial further research is needed to translate these perspectives into standardized and operationalizable assessment and intervention protocols.

With stronger methodological foundations, SARs may be integrated more effectively into rehabilitation programmes that extend beyond cognitive performance alone and sustain a holistic, experience-based approach to care and support.

## Figures and Tables

**Table 1:** Search strategies used for each database.

Database	String
Pubmed	(“Dementia”[MeSH] OR dementia[tiab] OR “Alzheimer Disease”[MeSH] OR Alzheimer*[tiab] OR “Cognitive Dysfunction”[MeSH] OR “Cognitive Decline”[tiab] OR “Cognitive Impairment”[tiab] OR “Mild Cognitive Impairment”[MeSH] OR “MCI”[tiab] OR “neurocognitive disorder”[tiab] OR “cognitive disorder”[tiab] OR “memory disorder”[tiab] OR “older adults”[tiab] OR elderly[tiab]) AND (“Cognitive Training”[MeSH] OR “Cognition”[MeSH:noexp] OR “Cognitive Therapy”[MeSH] OR “Neurological rehabilitation”[MeSH Terms] OR “Rehabilitation”[MeSH] OR “cognitive training”[tiab] OR “cognitive stimulation”[tiab] OR “cognitive intervention”[tiab] OR “cognitive exercise”[tiab] OR “cognitive rehabilitation”[tiab] OR “cognitive therapy”[tiab]) AND (“Robotics”[MeSH] OR robot*[tiab] OR “social robot”[tiab] OR “assistive robot”[tiab] OR “companion robot”[tiab] OR “care robot”[tiab] OR “therapeutic robot”[tiab] OR “socially assistive robot”[tiab] OR “social assistive robot”[tiab] OR SAR[tiab] OR “human-robot interaction”[tiab] OR HRI[tiab]) AND (systematic[sb]_OR “meta-analysis”[pt])
Springer, Wiley, and ACM digital library	(“Dementia” OR “Alzheimer Disease” OR “Alzheimer*” OR “cognitive dysfunction” OR “cognitive decline” OR “mild cognitive impairment” OR “MCI” OR “mild neurocognitive disorder*” OR “cognitive disability” OR “cognitive disorder*” OR “neurocognitive disorder*” OR “memory disorder*” OR “memory impairment” OR “aged” OR “older adults” OR “elderly”) AND (“cognitive training” OR “cognitive rehabilitation” OR “cognitive therapy” OR “neurological rehabilitation” OR “neurorehabilitation”) AND (“robot*” OR “socially assistive robot*” OR “social assistive robot*” OR “SAR” OR “assistive robot*” OR “companion robot*” OR “care robot*” OR “rehabilitation robot*” OR “therapeutic robot*” OR “social robot*” OR “human-robot interaction” OR “HRI” OR “robot-assisted intervention*” OR “robot intervention*” OR “robot-assisted cognitive training”) AND (“review” OR “systematic review” OR “meta-analysis” OR “meta analysis”)
IEEE Xplore	(“Dementia” OR “Alzheimer Disease” OR “Alzheimer” OR “cognitive dysfunction” OR “cognitive decline” OR “mild cognitive impairment” OR “MCI” OR “mild neurocognitive disorder” OR “cognitive disability” OR “cognitive disorder” OR “neurocognitive disorder” OR “memory disorder” OR “memory impairment” OR “aged” OR “older adults” OR “elderly”) AND (“cognitive training” OR “cognitive rehabilitation” OR “cognitive therapy” OR “neurological rehabilitation” OR “neurorehabilitation”) AND (“robot” OR “socially assistive robot” OR “social assistive robot” OR “SAR” OR “assistive robot” OR “companion robot” OR “care robot” OR “rehabilitation robot” OR “therapeutic robot” OR “social robot” OR “human-robot interaction” OR “HRI” OR “robot-assisted intervention” OR “robot intervention” OR “robot-assisted cognitive training”) AND (“review” OR “systematic review” OR “meta-analysis” OR “meta analysis”)
Scopus	(TITLE-ABS-KEY(“Dementia” OR “Alzheimer Disease” OR “Alzheimer*” OR “cognitive dysfunction” OR “cognitive decline” OR “mild cognitive impairment” OR “MCI” OR “mild neurocognitive disorder*” OR “cognitive disability” OR “cognitive disorder*” OR “neurocognitive disorder*” OR “memory disorder*” OR “memory impairment” OR “aged” OR “older adults” OR “elderly”)) AND ( TITLE-ABS-KEY(“cognitive training” OR “cognitive rehabilitation” OR “cognitive therapy” OR “neurological rehabilitation” OR “neurorehabilitation”)) AND ( TITLE-ABS-KEY(“robot*” OR “socially assistive robot*” OR “social assistive robot*” OR “SAR” OR “assistive robot*” OR “companion robot*” OR “care robot*” OR “rehabilitation robot*” OR “therapeutic robot*” OR “social robot*” OR “human-robot interaction” OR “HRI” OR “robot-assisted intervention*” OR “robot intervention*” OR “robot-assisted cognitive training”)) AND (TITLE-ABS-KEY(“review” OR “systematic review” OR “meta-analysis” OR “meta analysis”)) AND PUBYEAR > 2014 AND PUBYEAR < 2026.
ScienceDirect	(“dementia” OR “Alzheimer” OR “mild cognitive impairment”) AND (“social robot” OR “socially assistive robot” OR “robot therapy”) AND (“systematic review” OR “meta-analysis”)

**Table 2:** Characteristics of included systematic reviews and meta-analyses.

	Reference	Total Sample Size	Method of Analysis	Type of SARs Included	No. of Included Studies	Years Range of Publication	Outcomes (with Respective Measures and Indicators)	Findings by Outcome Domain	Heterogeneity
1	Moyle et al. [42].	N = 19	QLT	Telepresence robots with videoconferencing capability, including: Companionable robot Guide robot Giraff robot	4 QLT (interviews, observations, diaries).	2012–2014	Emotional response, Acceptability, and Usability: thematic analysis and observational sheets.	Emotional response: residents showed positive emotions, especially at the beginning and end of video calls. Users attributed human-like qualities to some robots, reflecting emotional acceptance. Acceptability and Usability: overall strong acceptance among patients, families, and staff. Participants found robots enjoyable, interesting, and useful. Family members appreciated reduced travel burden and reassurance from seeing loved ones.	N/A
2	Kang et al. [43].	N = 1250	QLT	PARO	8 RCTs	2003–2018	Cognition: GDS, MMSE, sMMSE, ACE-III. Affect/Emotion: OERS, facial expression coding, mood states. Social interaction/engagement: Observational coding (talking, visual/verbal engagement). Neuropsychiatric Symptoms: APADEM-NH, AES, GDS, CSDD, RAID, BARS, CMAI-SF, NPI-Q, NPI-NH, AWS, Quality of Life: QOL-AD, QUALID. Physiological: blood pressure, heart rate, salivary and hair. cortisol, GSR, motor activity and sleep via wearables. Medication Use: Psychotropic, pain, dementia-related medications.	Cognition did not show significant change. Affect and Social interaction: increased pleasure, smiling, positive mood, verbal/visual engagement, and social interaction with staff/others. Neuropsychiatric symptoms: reductions observed in agitation, depression, anxiety, apathy in some studies, though effects varied by measure and study. Quality of life: improved across studies measuring it, including late-stage dementia. Physiological: some reduction in heart rate and stress markers in isolated studies; cortisol results inconsistent; decreased daytime motor activity in one study. Medical treatment: reduction in psychotropic and pain medication use in two studies; no change in dementia-related medication in one study.	N/A
3	Leng et al. [38].	N = 502	QNT	PARO	6 RCTs	2008–2013	Cognitive function: MMSE; GDS. Agitation: CMAI-SF; BARS. Anxiety and apathy: RAID; APADEM-NH. Depression: CSDD; GDS. Quality of life: QOL-AD; QUALID.	Cognitive function: no significant change (SMD = 0.02; 95% CI −0.29 to 0.34; *p* = 0.88). Agitation: significant beneficial effect (SMD = −0.37; 95% CI −0.64, −0.09; *p* = 0.008). Anxiety and apathy: significant improvement (SMD = −0.42; 95% CI −0.72 to −0.11; *p* = 0.008). Depression: significant beneficial effect (SMD = −0.35; 95% CI −0.65 to −0.04; *p* = 0.03). Quality of life: no significant effect (SMD = 0.19; 95% CI −0.64 to 1.01, *p* = 0.66).	Cognitive function: I^2^ = 0%. Agitation: I^2^ = 28%. Anxiety and apathy: I^2^ = 0%. Depression: I^2^ = 66%. Quality of life: I^2^ = 85%.
4	Pu et al. [41].	N = 1042	QNT/QLT	PARO AIBO NAO	11 RCTs	2008–2017	Cognitive level: MMSE; GDS; ACE. Agitation: CMAI-SF; BARS, Anxiety: RAID. Depression: CSDD; GDS. Neuropsychiatric symptoms: NPI/NPI-Q, Quality of life: QOL-AD; QUALID.	Cognitive level: no significant change (SMD = 0.04; 95% CI −0.33 to 0.26). Agitation: no significant change (SMD = −0.20; 95% CI −0.57 to 0.17). Anxiety: no significant change (SMD = 2.8; 95% CI −1.58 to 7.18). Depression and apathy: no significant improvement (SMD = 0.06; 95% CI −0.17 to 0.29) and (SMD = 0.00; 95% CI −0.34 to 0.35). Neuropsychiatric symptoms: no significant change (SMD = 0.09; 95% CI −0.27 to 0.45). Quality of life: no significant change (SMD = 0.21; 95% CI −0.47 to 0.88). Engagement, Social Interaction, and Loneliness: qualitative data shows positive results. Medication: lower psychotropic or pain-medication use.	Cognitive level: I^2^ = 0%. Agitation: I^2^ = 27%. Anxiety: I^2^ = N/A. Depression and apathy: I^2^ = 0%. Neuropsychiatric symptoms: I^2^ = 0%. Quality of life: I^2^ = N/A.
5	Vogan et al. [31].	ns	QLT	PARO, CuDDler. NAO, Pepper, Sil-bot, Kabochan, Mero.	11	2007–2019	Cognitive function: MMSE; severe MMSE; MoCA; GDS; ACE; SWM. Social-emotional response: CSDD; RAID; NPI; APS; CMAI-SF; QUALID. User Preference and Acceptability: QUS.	Cognitive domain: Robot-assisted training improved executive function, verbal scores, and judgement. One primary study also associated it with reduced cortical thinning. Social-emotional response: SARs led to reduced stress, anxiety, agitation, and loneliness, and improved mood, social engagement, communication, and emotional state. User Preference and Acceptability: generally positive acceptance of SARs among older adults. One primary study shows preference for Pepper and humanoid robots.	N/A
6	Ghafurian et al. [32].	ns	QLT	PARO, AIBO, Robocat, NeCoRo, JustoCat, HUG, Probo. NAO, Pepper, Kabochan, Ifbot Giraff, Telenoid (telepresence robot).	53	2002–2019	Robot application areas: (1) ADLs. (2) Companionship. (3) Engagement. (4) Health Guidance. (5) Therapy.	Engagement effects often extend to social interaction between users, not only with robots. SARs provide diet-related reminders and improve quality of care by reducing caregiver stress, as SAR interventions often eased care burden. Improved mood, well-being, and quality of life.	N/A
7	Lu et al. [44].	Agitation: (N = 214). Depression: (N = 170). Quality of life: (N = 161).	QNT	PARO. NAO.	13	2013–2019	Agitation: BARS, CMAI-SF. Depression: CSDD, GDS. Quality of life: QoL-AD, QUALID.	Agitation: significant reduction (SMD = −0.37; 95% CI −0.64 to −0.10; *p* < 0.01). Depression: no significant pooled effect (SMD = −0.10; 95% CI −0.52 to 0.31; *p* = 0.23). However, meta-regression showed longer session length and higher weekly exposure time improved depressive symptoms. Quality of life: no significant effect (SMD = 0.17; 95% CI: −0.15 to 0.48; *p* = 0.63).	Agitation: I^2^ = 0%. Depression: I^2^ = 97%. Quality of life: I^2^ = 64%.
8	Ong et al. [34].	N = 1256	QNT	PARO. NAO. Telenoid R3. Lifelike baby dolls. Cota and PALRO.	14	2011–2019	Cognitive status: MMSE; RUDAS; GDS; sMMME. Agitation: CMAI/CMAI-SF; BARS. Anxiety: RAID. Depression: CSDD; GDS. Quality of life: QUALID, QoL-AD. Social Interaction: observational coding scales (facial/physical/verbal engagement).	Cognitive status: no significant change (SMD = 0.05; 95% CI −0.22 to 0.32; *p* = 0.71). Agitation: significant improvement (SMD −0.38; 95% CI −0.66 to 0.09; *p* = 0.01). Anxiety: no significant improvement (SMD = 0.15; 95% CI −0.15 to 0.46; *p* = 0.71). Depression: no significant change (SMD = 0.05; 95% CI −0.38 to 0.49; *p* = 0.81). Social interaction: significant change (SMD = 0.49; 95% CI 0.01 to 0.97; *p* = 0.04). Positive mood: (SMD = 0.43; 95% CI 0.21 to 0.64; *p* <0.0001). Quality of life: no significant change (SMD = 0.09; 95% CI −0.28 to 0.46; *p* = 0.64). Medication usage: (SMD = 0.50; 95% CI 0.92 to 0.09; *p* = 0.02).	Cognitive function: I^2^ = 0%. Agitation: I^2^ = 32%. Anxiety: I^2^ = 41%. Depression: I^2^ = 62%. Social interaction: I^2^ = 49%. Positive mood: I^2^ = 0%. Quality of life: I^2^ = 31%. Medication usage: I^2^ = 24%.
9	Saragih et al. [39].	N = 1684	QNT	PARO. Kabochan, NAO. Bomy (table-top robot).	15 RCTs (including 3 cluster-RCTs and 5 pilot-RCTs)	2013–2021	Cognitive function: MMSE; MoCA Agitation: CMAI-SF; BARS. Anxiety: GAI; RAID; mood-state subscales. Depression: CSDD; GDS. Neuropsychiatric symptoms: NPI/NPI-Q; SWM. Sleep: Daytime sleep hours (from activity/sleep logs). Quality of Life: QOL-AD; QUALID.	Cognitive function: no significant change (SMD = 0.16; 95% CI −0.08 to 0.40; *p* = 0.20). Agitation: no significant change (SMD = −0.09; 95% CI −0.22 to 0.33; *p* = 0.15). Anxiety: no significant change (SMD = −0.07; 95% CI −0.42 to 0.28; *p* = 0.68). Depression: significant improvement (SMD = −0.35; 95% CI −0.69 to −0.02; *p* = 0.04). Neuropsychiatric symptoms: no significant change (SMD = 0.16; 95% CI −0.29 to 0.61; *p* = 0.49). Daytime sleep: less daytime sleep and better sleep pattern (SMD = −0.31; 95% CI −0.55 to 0.07; *p* = 0.01). Quality of life: no significant change (SMD = 0.24; 95% CI −0.23 to 0.70; *p* = 0.31).	Cognitive function: I^2^ = 0%. Agitation: I^2^ = 0%. Anxiety: I^2^ = 43.11%. Depression: I^2^ = 0%. Neuropsychiatric symptoms: I^2^ = 63.92%. Sleep: I^2^ = 0%. Quality of life: I^2^ = 65.94%.
10	Lee et al. [33].	N = 575	QNT	PARO. NAO, Sil-bot. Silver-Care robot, ×10 ActiveHome kit (smart-home system).	9 total (6 RCTs, 3 quasi-experimental studies).	2007–2021	Cognitive function: MMSE.	Cognitive function: significant improvement (*g* = 0.43; 95% CI −0.04 to 0.90).	Cognitive function: I^2^ = 86%.
11	Yu et al. [45].	N = 1750	QNT/QLT	Companion robots as PARO, robot cats/dogs, and humanoid companions). Telepresence robots. Home-care assistive robots. Multifunctional robots.	66 total (RCTs, non RCTs, qualitative studies).	2004–2022	Cognition: ACE; MMSE; RUDAS. Neuropsychiatric symptoms: NPI-Q, Agitation: CMAI/CMAI-SF; BARS. Anxiety: RAID. Depression: CSDD. Apathy: AES; AI. Quality of life: QUALID; QoL-AD. Feasibility and Acceptability: SUS, attrition, attendance, observed engagement/affect, interviews with patients, caregivers, staff.	Cognition: no significant change (SMD = 0.03; 95% CI 0.32 to 0.38). Agitation: no significant change (SMD = −0.25; 95% CI 0.57–0.06). Anxiety: no significant change (SMD = 0.24; 95% CI 0.85 to 1.33). Depression: no significant improvement (SMD = 0.08; 95% CI 0.52 to 0.69). Apathy: no significant improvement (SMD = 0.14; 95% CI 0.29 to 0.58). Neuropsychiatric symptoms: no significant change (SMD = −0.01; 95% CI 0.32 to 0.29) Quality of life: no significant change (SMD = 0.05; 95% CI 0.52 to 0.42). Feasibility: SAR interventions were practical to deliver and useful for stimulating interaction, although challenges include costs and staff training. Acceptability: patients enjoyed interacting with SARs, particularly companion robots as PARO. Telepresence robots were less acceptable to users with moderate–severe cognitive impairment due to interface complexity.	Cognition: I^2^ = 0%. Agitation: I^2^ = 0%. Anxiety: I^2^ = 86%. Depression: I^2^ = 75%. Apathy: I^2^ = 0% Neuropsychiatric symptoms: I^2^ = 0%. Quality of life: I^2^ = 43%.
12	Figliano et al. [15].	N = 840	QLT	NAO, PEPPER, SILBOT, MERO, BOMY, RYAN, NADINE, MATILDA, JAMES, JACK, SOPHIE (humanoid robots—15 studies). PARO, AIBO (zoomorphic robots—3 studies). Mixed comparison (1 study): NAO + PARO. All used the Wizard-of-Oz method (human-controlled interaction).	19 total (9 RCTs, 9 qualitative/observational studies, and 1 Pilot study).	2000–2022	Cognitive function: MMSE; MoCA; CANTAB; SNSB; ADAS-Cog; MRI imaging (in one study). Emotional and social engagement: QOL SF-8; STAI; PHQ-9, NPI; OERS); MPES; behavioural observation (frequency of social interaction, positive expressions, verbal communication). Acceptability: ns.	Cognitive function: SAR-assisted interventions improved memory and executive functions, comparable to traditional clinician-led training. Emotional response and social engagement: zoomorphic robots reduced loneliness and increased positive affect and social interaction; humanoid robots fostered engagement, conversation, and emotional responses. Overall, SARs enhanced psychological well-being and social engagement in group activities. Acceptability: high acceptability across all age and cognitive levels.	N/A
13	Hsieh et al. [37].	N = 1712	QNT	ns	14 RCTs (9 included in meta-analysis).	2011–2022	Depression: GDS; CSDD. Agitation: CMAI-SF. Anxiety: RAID. Neuropsychiatric symptoms: NPI/NPI-Q Quality of life: QoL-AD; QUALID. Positive emotion: OERS. Social interaction: behavioural observation (frequency of social interaction, cooperation, verbal communication). Activity/sleep: SenseWear Armband data.	Depression: significant reduction (MD = 4.73; 95% CI 4.54 to 4.93; *p* < 0.001); effects significant for clinician-rated CSDD only. Agitation: no significant change (MD = −2.07; 95% CI −4.93 to 0.78; *p* = 0.15). Anxiety: significant reduction (MD = 2.80; 95% CI 2.70 to 2.90; *p* < 0.001). Neuropsychiatric: non-significant (MD = −0.63; 95% CI −2.24 to 0.97; *p* = 0.44). Quality of life: non-significant (MD = −0.38; 95% CI −1.92 to 1.16; *p* = 0.63). Positive emotion: significant increase (MD = −2.44; 95% CI −3.91 to −0.97; *p* < 0.01). Social interaction: significant improvement (MD = −5.07; 95% CI −7.80 to −2.34; *p* < 0.001).	Depression: I^2^ = 97%. Agitation: 44% Anxiety: I^2^ = 61%. Neuropsychiatric symptoms: I^2^ = 0%. Quality of life: I^2^ = 47% Positive emotion: I^2^ = 70%. Social interaction: I^2^ = 74%.
14	Noh & Shim [35].	N = 1191	QNT	PARO—most common. NAO, Sil-bot, Kabochan. Bomy.	10 studies in the systematic review. 9 included in the meta-analysis (5 RCTs, 2 cluster-RCTs).	2015–2021	Cognitive function: MMSE-DS; MoCA; ACE; Severe MMSE; CANTAB. Agitation: CMAI; BARS. Anxiety: RAID; GAI. Depression: GDS; CSDD. Neuropsychiatric symptoms: NPI. Quality of life: QoL-AD; QUALID.	Cognitive function: no significant improvement overall (*g* = 0.04). Agitation: significant reduction (*g* = −0.31). Individual SAR sessions more effective than group sessions. Anxiety: significant reduction (*g* = −0.43). Pet-type robots produced medium-sized improvements. Depression: non-significant reduction overall (*g* = −0.27, *p* ≈ 0.05). Group-based SAR showed greater benefit. Neuropsychiatric symptoms: no significant change (*g* = −0.05). Quality of life: no significant improvement (*g* = 0.02).	Cognitive function: I^2^ = 0%. Agitation: I^2^ = 28.92%. Anxiety: I^2^ = 0% Depression: I^2^ = 0%. Neuropsychiatric symptoms: I^2^ = 0%. Quality of life: I^2^ = 10.45%.
15	Yen et al. [36].	N = 918	QNT	PARO, AIBO. Pepper, Kobochan.	8 RCTs	2008–2022	Depression: GDS; CSDD; video-based observation of sadness. Loneliness: UCLA-LS.	Depression: significant improvement (*g* = −0.91; *p* = 0.026), with stronger effects when interventions were ≥12 weeks and group-based. Loneliness: significant improvement (*g* = −1.21; *p* = 0.023).	Depression: I^2^ = 94%. Anxiety: I^2^ = 79%.
16	Fan et al. [40].	N = 705	QNT	PARO NAO	15 RCTs (12 in meta-analysis)	2015–2023	Cognitive function: MMSE; Severe MMSE. Neuropsychiatric symptoms: ns. Agitation: ns. Anxiety: ns. Depression: ns. Quality of life: ns. Physical activity and sleep: Sense Wear Armband data.	Cognitive function: no significant improvement (SMD = 0.09; 95% CI −0.09 to 0.26; *p* = 0.46). Agitation: significant reduction (SMD = −0.36; 95% CI −0.56 to −0.17; *p* < 0.001). Anxiety: significant reduction (WMD = −1.93; 95% CI −3.13 to −0.72; *p* = 0.002). Depression: not significant overall (SMD = −0.20; *p* = 0.26), but improved in long-duration interventions (≥12 weeks). Neuropsychiatric symptoms: no significant effect (SMD = −0.09; 95% CI −0.35 to 0.16; *p* = 0.46). Quality of life: no significant change (SMD = 0.05; *p* = 0.77). Physical activity and sleep: slight reduction in daytime lying time (WMD = −0.48 h; *p* = 0.02), but no differences in night-time activity or step count.	Cognitive function: I^2^ = 0. Agitation: I^2^ = 0%. Anxiety: I^2^ = 0% Depression: I^2^ = 67.8%. Neuropsychiatric symptoms: I^2^ = 50.2%. Quality of life: I^2^ = 66.1%. Physical activity and sleep: I^2^ = 0%.

Legend: ACE—Addenbrooke’s Cognitive Examination; ACE-III—Addenbrooke’s Cognitive Examination—III; ADLs—Activities of Daily Living; ADAS-Cog—Alzheimer’s Disease Assessment Scale—Cognitive Subscale; AES—Apathy Evaluation Scale; AI—Apathy Inventory; APADEM-NH—Apathy in Institutionalized Elderly with Neurocognitive Disorders (Nursing Home version); APS—Activity Participation Scale; AWS—AWS; BARS—Brief Agitation Rating Scale; CANTAB—Cambridge Neuropsychological Test Automated Battery; CMAI—Cohen–Mansfield Agitation Inventory; CMAI-SF—Cohen–Mansfield Agitation Inventory—Short Form; CG—Caregivers; CSDD—Cornell Scale for Depression in Dementia; GAI—Geriatric Anxiety Inventory; GDS—Global Deterioration Scale; GDS (Depression)—Geriatric Depression Scale; GDSC—Global Deterioration Scale; GSR—Galvanic Skin Response; K-CMAI—Korean version of the Cohen-Mansfield Agitation Inventory; LTC—Long-Term Care; MMSE—Mini-Mental State Examination; MMSE-DS—Mini-Mental State Examination—Dysphasia/Downscaled version; MMSE-K—Mini-Mental State Examination—Korean version; MoCA—Montreal Cognitive Assessment; MPES—Menorah Park Engagement Scale; MRI—Magnetic Resonance Imaging; NAO—Multifunctional humanoid robot; NPI—Neuropsychiatric Inventory; NPI-NH—Neuropsychiatric Inventory—Nursing Home version; NPI-Q—Neuropsychiatric Inventory—Questionnaire; OERS—Observed Emotion Rating Scale; PHQ-9—Patient Health Questionnaire-9; QUALID—Quality of Life in Late-Stage Dementia; QOL-AD—Quality of Life in Alzheimer’s Disease; QOL SF-8—Short Form-8 Quality of Life; QUS—Qualitative Usability Study; RAID—Rating Anxiety in Dementia; RUDAS—Rowland Universal Dementia Assessment Scale; sMMSE—Standardized Mini-Mental State Examination; Severe MMSE—Severe Mini-Mental State Examination; SNSB—Seoul Neuropsychological Screening Battery; STAI—State-Trait Anxiety Inventory; SUS—System Usability Scale; SWM—Spatial Working Memory; UCLA-LS—UCLA Loneliness Scale.

**Table 3:** Vote counting by direction of effect reported within included reviews.

Outcome	n (Studies)	u (Favoured SARs)	*p* = u/n (95% CI)	Sign-Test *p*-Value	Direction of Evidence
Cognitive function	11	3	0.27 (0.09–0.56)	0.22	No consistent evidence of benefit
Anxiety	10	6	0.60 (0.31–0.83)	0.75	Trend toward benefit, but not significant
Agitation	11	7	0.64 (0.35–0.85)	0.55	Trend toward benefit, but not significant
Depression	11	5	0.45 (0.21–0.72)	1.00	Mixed results, no consistent evidence of benefit
Neuropsychiatric symptoms	7	0	0.00 (0.00–0.35)	0.016	Consistent evidence of no benefit
Emotional response and social engagement	8	8	1.00 (0.67–1.00)	0.007	Consistent evidence of benefit
Quality of life	11	2	0.18 (0.05–0.47)	0.065	Trend toward no benefit, but not significant

## Data Availability

Materials are available from the corresponding author upon request.

## Supplementary Materials

The following supporting information can be downloaded at: https://www.mdpi.com/article/10.3390/healthcare14010066/s1, Table S1. Included studies with respective sources from the search for systematic reviews and meta-analyses. Table S2. Excluded studies with reasons. Table S3. AMSTAR-2 results for individual studies included in the umbrella review. Table S4. ROBIS results for individual studies included in the umbrella review. Table S5. Citation matrix of all primary studies (rows) included for each review (columns) used to calculate the CCA.
