# Supplementary material for: From Social Robotics to Ecological Cognitive Care: An Enaction-Based Umbrella Review on Neurocognitive Disorders
Source: Healthcare (Basel). 2025 Dec 26;14(1):66. doi: 10.3390/healthcare14010066 (PMC12786088; doi:10.3390/healthcare14010066)
Supplement: Supplementary file 1 [file healthcare-14-00066-s001.zip › healthcare-4031133-supplementary.pdf]

PRISMA checklist

Section and Topic	Item #	Checklist item	Location where item is reported
**TITLE**			
Title	1	Identify the report as a systematic review.	Title
**ABSTRACT**			
Abstract	2	See the PRISMA 2020 for Abstracts checklist.	See abstract checklist
**INTRODUCTION**			
Rationale	3	Describe the rationale for the review in the context of existing knowledge.	Introduction section 1.2
Objectives	4	Provide an explicit statement of the objective(s) or question(s) the review addresses.	Introduction section 1.2
**METHODS**			
Eligibility criteria	5	Specify the inclusion and exclusion criteria for the review and how studies were grouped for the syntheses.	Method section 2.2
Information sources	6	Specify all databases, registers, websites, organisations, reference lists and other sources searched or consulted to identify studies. Specify the date when each source was last searched or consulted.	Method section 2.3
Search strategy	7	Present the full search strategies for all databases, registers and websites, including any filters and limits used.	Method section 2.3
Selection process	8	Specify the methods used to decide whether a study met the inclusion criteria of the review, including how many reviewers screened each record and each report retrieved, whether they worked independently, and if applicable, details of automation tools used in the process.	Method section 2.4
Data collection process	9	Specify the methods used to collect data from reports, including how many reviewers collected data from each report, whether they worked independently, any processes for obtaining or confirming data from study investigators, and if applicable, details of automation tools used in the process.	Method section 2.5

Data items	10a	List and define all outcomes for which data were sought. Specify whether all results that were compatible with each outcome domain in each study were sought (e.g. for all measures, time points, analyses), and if not, the methods used to decide which results to collect.	Method section 2.5
	10b	List and define all other variables for which data were sought (e.g. participant and intervention characteristics, funding sources). Describe any assumptions made about any missing or unclear information.	Method section 2.5
Study risk of bias assessment	11	Specify the methods used to assess risk of bias in the included studies, including details of the tool(s) used, how many reviewers assessed each study and whether they worked independently, and if applicable, details of automation tools used in the process.	Method section 2.6
Effect measures	12	Specify for each outcome the effect measure(s) (e.g. risk ratio, mean difference) used in the synthesis or presentation of results.	Table 2
Synthesis methods	13a	Describe the processes used to decide which studies were eligible for each synthesis (e.g. tabulating the study intervention characteristics and comparing against the planned groups for each synthesis (item #5)).	Table 2 caption
	13b	Describe any methods required to prepare the data for presentation or synthesis, such as handling of missing summary statistics, or data conversions.	Method section 2.7
	13c	Describe any methods used to tabulate or visually display results of individual studies and syntheses.	Table 2 caption
	13d	Describe any methods used to synthesize results and provide a rationale for the choice(s). If meta-analysis was performed, describe the model(s), method(s) to identify the presence and extent of statistical heterogeneity, and software package(s) used.	Method section 2.7
	13e	Describe any methods used to explore possible causes of heterogeneity among study results (e.g. subgroup analysis, meta-regression).	N/A
	13f	Describe any sensitivity analyses conducted to assess robustness of the synthesized results.	N/A
Reporting bias assessment	14	Describe any methods used to assess risk of bias due to missing results in a synthesis (arising from reporting biases).	N/A
Certainty assessment	15	Describe any methods used to assess certainty (or confidence) in the body of evidence for an outcome.	Supplementary Material S3-S4
**RESULTS**			
Study selection	16a	Describe the results of the search and selection process, from the number of records identified in the search to the number of studies included in the review, ideally using a flow diagram.	Supplementary Material S1
	16b	Cite studies that might appear to meet the inclusion criteria, but which were excluded, and explain why they were excluded.	Supplementary Material S2

Study characteristics	17	Cite each included study and present its characteristics.	Table 2
Risk of bias in studies	18	Present assessments of risk of bias for each included study.	Supplementary Material Tables S3-S4
Results of individual studies	19	For all outcomes, present, for each study: (a) summary statistics for each group (where appropriate) and (b) an effect estimate and its precision (e.g. confidence/credible interval), ideally using structured tables or plots.	Table 2
Results of syntheses	20a	For each synthesis, briefly summarise the characteristics and risk of bias among contributing studies.	Results section 3.3
	20b	Present results of all statistical syntheses conducted. If meta-analysis was done, present for each the summary estimate and its precision (e.g. confidence/credible interval) and measures of statistical heterogeneity. If comparing groups, describe the direction of the effect.	Results section 3.3
	20c	Present results of all investigations of possible causes of heterogeneity among study results.	Results section 3.3
	20d	Present results of all sensitivity analyses conducted to assess the robustness of the synthesized results.	N/A
Reporting biases	21	Present assessments of risk of bias due to missing results (arising from reporting biases) for each synthesis assessed.	N/A
Certainty of evidence	22	Present assessments of certainty (or confidence) in the body of evidence for each outcome assessed.	Table 3
**DISCUSSION**			
Discussion	23a	Provide a general interpretation of the results in the context of other evidence.	Discussion section 4
	23b	Discuss any limitations of the evidence included in the review.	Conclusion section 5
	23c	Discuss any limitations of the review processes used.	Conclusion section 5
	23d	Discuss implications of the results for practice, policy, and future research.	Conclusion section 5
**OTHER INFORMATION**			

Registration and protocol	24a	Provide registration information for the review, including register name and registration number, or state that the review was not registered.	Abstract and method section
	24b	Indicate where the review protocol can be accessed, or state that a protocol was not prepared.	Abstract and method section
	24c	Describe and explain any amendments to information provided at registration or in the protocol.	Method section 2.1
Support	25	Describe sources of financial or non-financial support for the review, and the role of the funders or sponsors in the review.	Funding section
Competing interests	26	Declare any competing interests of review authors.	Conflicts of interest section
Availability of data, code and other materials	27	Report which of the following are publicly available and where they can be found: template data collection forms; data extracted from included studies; data used for all analyses; analytic code; any other materials used in the review.	Dataset available on request from the authors

PRISMA checklist for abstract

Section and Topic	Item #	Checklist item	Reported (Yes/No)
**TITLE**			
Title	1	Identify the report as a systematic review.	Yes
**BACKGROUND**			
Objectives	2	Provide an explicit statement of the main objective(s) or question(s) the review addresses.	Yes
**METHODS**			
Eligibility criteria	3	Specify the inclusion and exclusion criteria for the review.	No

Information sources	4	Specify the information sources (e.g. databases, registers) used to identify studies and the date when each was last searched.	Yes
Risk of bias	5	Specify the methods used to assess risk of bias in the included studies.	No
Synthesis of results	6	Specify the methods used to present and synthesise results.	Yes
**RESULTS**			
Included studies	7	Give the total number of included studies and participants and summarise relevant characteristics of studies.	No
Synthesis of results	8	Present results for main outcomes, preferably indicating the number of included studies and participants for each. If meta-analysis was done, report the summary estimate and confidence/credible interval. If comparing groups, indicate the direction of the effect (i.e. which group is favoured).	Yes
**DISCUSSION**			
Limitations of evidence	9	Provide a brief summary of the limitations of the evidence included in the review (e.g. study risk of bias, inconsistency and imprecision).	Yes
Interpretation	10	Provide a general interpretation of the results and important implications.	Yes
**OTHER**			
Funding	11	Specify the primary source of funding for the review.	No
Registration	12	Provide the register name and registration number.	Yes

Supplementary Table S1. Included studies with respective sources from the search for systematic reviews and meta-analyses

Database	References
PubMed	1. Leng, M., Liu, P., Zhang, P., Hu, M., Zhou, H., Li, G., ... & Chen, L. (2019). Pet robot intervention for people with dementia: A systematic review and meta-analysis of randomized controlled trials. Psychiatry research, 271, 516-525.<br>2. Yu, C., Sommerlad, A., Sakure, L., & Livingston, G. (2022). Socially assistive robots for people with dementia: systematic review and meta-analysis of feasibility, acceptability and the effect on cognition, neuropsychiatric symptoms and quality of life. Ageing research reviews, 78, 101633.
"Similar Article" from PubMed	1. Hsieh, C. J., Li, P. S., Wang, C. H., Lin, S. L., Hsu, T. C., & Tsai, C. M. T. (2023). Socially assistive robots for people living with dementia in long-term facilities: a systematic review and meta-analysis of randomized controlled trials. Gerontology, 69(8), 1027-1042.<br>2. Lee, H., Chung, M. A., Kim, H., & Nam, E. W. (2022). The effect of cognitive function health care using artificial intelligence robots for older adults: systematic review and meta-analysis. JMIR aging, 5(2), e38896.<br>3. Lu, L. C., Lan, S. H., Hsieh, Y. P., Lin, L. Y., Lan, S. J., & Chen, J. C. (2021). Effectiveness of companion robot care for dementia: a systematic review and meta-analysis. Innovation in aging, 5(2), igab013.<br>4. Noh, D., & Shim, M. S. (2023, August). Effectiveness of robot interventions for cognitive and psychological outcomes among older adults with cognitive impairment: A meta-analysis. In Healthcare (Vol. 11, No. 16, p. 2341). MDPI.<br>5. Pu, L., Moyle, W., Jones, C., & Todorovic, M. (2019). The effectiveness of social robots for older adults: a systematic review and meta-analysis of randomized controlled studies. The gerontologist, 59(1), e37-e51.
Scopus	1. Figliano, G., Manzi, F., Tacci, A. L., Marchetti, A., & Massaro, D. (2023). Ageing society and the challenge for social robotics: A systematic review of Socially Assistive Robotics for MCI patients. Plos one, 18(11), e0293324.
ScienceDirect	1. Fan, W., Zhao, R., Liu, X., & Ge, L. (2025). Intelligent robot interventions for people with dementia: Systematic review and meta-analysis of randomized controlled trials. Journal of Medical Internet Research, 27, e59892.

	2. Kang, H. S., Makimoto, K., Konno, R., & Koh, I. S. (2020). Review of outcome measures in PARO robot intervention studies for dementia care. Geriatric Nursing, 41(3), 207-214.<br>3. Ong, Y. C., Tang, A., & Tam, W. (2021). Effectiveness of robot therapy in the management of behavioural and psychological symptoms for individuals with dementia: A systematic review and meta-analysis. Journal of Psychiatric Research, 140, 381-394.<br>4. Yen, H. Y., Huang, C. W., Chiu, H. L., & Jin, G. (2024). The effect of social robots on depression and loneliness for older residents in long-term care facilities: a meta-analysis of randomized controlled trials. Journal of the American Medical Directors Association, 25(6).
Wiley	1. Saragih, I. D., Tonapa, S. I., Sun, T. L., Chia-Ju, L., & Lee, B. O. (2021). Effects of robotic care interventions for dementia care: A systematic review and meta-analysis randomised controlled trials. Journal of Clinical Nursing, 30(21-22), 3139-3152.
IEEE Xplore	1. Vogan, A. A., Alnajjar, F., Gochoo, M., & Khalid, S. (2020). Robots, AI, and cognitive training in an era of mass age-related cognitive decline: a systematic review. Ieee Access, 8, 18284-18304.
ACM Digital Library	1. Ghafurian, M., Hoey, J., & Dautenhahn, K. (2021). Social robots for the care of persons with dementia: a systematic review. ACM Transactions on Human-Robot Interaction (THRI), 10(4), 1-31.

Supplementary Table S2. Excluded studies with reasons

Reasons of exclusion	References
Mixed populations (e.g., included healthy older adults or non-NCD clinical groups)	1. Alnajjar, F., Khalid, S., Vogan, A. A., Shimoda, S., Nouchi, R., & Kawashima, R. (2019). Emerging cognitive intervention technologies to meet the needs of an aging population: a systematic review. Frontiers in Aging Neuroscience, 11, 291.<br>2. Robledo-Castro, C., Castillo-Ossa, L. F., & Corchado, J. M. (2023). Artificial cognitive systems applied in executive function stimulation and rehabilitation programs: a systematic review. Arabian journal for science and engineering, 48(2), 2399-2427.

	3. Scoglio, A. A., Reilly, E. D., Gorman, J. A., & Drebing, C. E. (2019). Use of social robots in mental health and well-being research: systematic review. Journal of medical Internet research, 21(7), e13322.<br>4. Yuan, F., Klavon, E., Liu, Z., Lopez, R. P., & Zhao, X. (2021). A systematic review of robotic rehabilitation for cognitive training. Frontiers in Robotics and AI, 8, 605715.
Mixed or non-SAR interventions (e.g., combined with VR, tablets, or other technologies)	1. Lee-Cheong, S., Amanullah, S., & Jardine, M. (2022). New assistive technologies in dementia and mild cognitive impairment care: A PubMed review. Asian Journal of Psychiatry, 73, 103135.<br>2. Loveys, K., Prina, M., Axford, C., Domènec, Ò. R., Weng, W., Broadbent, E., ... & Thiyagarajan, J. A. (2022). Artificial intelligence for older people receiving long-term care: a systematic review of acceptability and effectiveness studies. The Lancet Healthy Longevity, 3(4), e286-e297.<br>3. Morales, D. M., Arenas, D. A. M., & Martínez, D. A. L. (2023). Working memory training with technological innovation in older adults with mild neurocognitive disorder: A systematic review using ToS (Tree of Science) methodology. Mediterranean Journal of Clinical Psychology, 11(3).<br>4. Seok, J. W., Shin, J., Kang, B., Lee, H., Cho, E., & Lee, K. H. (2022). Non-pharmacological interventions using information and communication technology for behavioral and psychological symptoms of dementia: A systematic review and meta-analysis protocol. Journal of Advanced Nursing, 78(1), 282-293.
Focused on technical development of SAR	1. Mohebbi, A. (2020). Human-robot interaction in rehabilitation and assistance: a review. Current Robotics Reports, 1(3), 131-144.<br>2. Tamantini, C., Umbrico, A., & Orlandini, A. (2025). Automated planning and scheduling in robot-aided rehabilitation: a review. Journal of NeuroEngineering and Rehabilitation, 22(1), 180.<br>3. Nicora, G., Pe, S., Santangelo, G., Billeci, L., Aprile, I. G., Germanotta, M., ... & Quaglini, S. (2025). Systematic review of AI/ML applications in multi-domain robotic rehabilitation: trends, gaps, and future directions. Journal of NeuroEngineering and Rehabilitation, 22(1), 79.<br>4. Lima, M. R., Wairagkar, M., Gupta, M., y Baena, F. R., Barnaghi, P., Sharp, D. J., & Vaidyanathan, R. (2021). Conversational affective social robots for ageing and dementia support. IEEE Transactions on Cognitive and Developmental Systems, 14(4), 1378-1397.<br>5. Pagliarini, L., & Lund, H. H. (2016). Redefining robot based technologies for elderly people assistance: a survey. Journal of Robotics, Networking and Artificial Life, 3(1), 28-32.<br>6. Rasa, A. R. (2024). Artificial intelligence and its revolutionary role in physical and mental rehabilitation: a review of recent advancements. BioMed Research International, 2024(1), 9554590.

	7. Yu, J., Yao, Y., Feng, R., Liang, T., Wang, W., & Li, J. (2023). A review of the text-to-speech synthesizer for human robot interaction for patients with Alzheimer's disease. Digital Medicine, 9(4), e00011.
Insufficient or non-extractable data for synthesis	1. Costanzo, M., Smeriglio, R., & Di Nuovo, S. (2024). New technologies and assistive robotics for elderly: A review on psychological variables. Archives of Gerontology and Geriatrics plus, 1(4), 100056.<br>2. Leung, C., Wong, K. C., So, W. W., Tse, Z. C., Li, D., Cao, Y., & Shum, D. H. (2022). The application of technology to improve cognition in older adults: A review and suggestions for future directions. PsyCh journal, 11(4), 583-599.<br>3. Rohrer, C., Ben Souissi, S., & Kurpicz-Briki, M. (2025). Systematic review of recent years: machine learning-based interactive therapy for people suffering from dementia. Artificial Intelligence Review, 58(3), 78.<br>4. Shishehgar, M., Kerr, D., & Blake, J. (2018). A systematic review of research into how robotic technology can help older people. Smart Health, 7, 1-18.<br>5. Vandemeulebroucke, T., De Casterlé, B. D., & Gastmans, C. (2018). The use of care robots in aged care: A systematic review of argument-based ethics literature. Archives of gerontology and geriatrics, 74, 15-25.<br>6. Yang, Y., & Zhou, J. (2025, May). Assistive Robots in Older Adult Care: A Systematic Review and Prospects. In International Conference on Human-Computer Interaction (pp. 186-202). Cham: Springer Nature Switzerland.
Did not report relevant outcomes	1. He, X., Hao, J., Song, Y., Cao, H., Chen, Y., & Yang, H. (2023). Effectiveness of non-pharmacological interventions for sleep disturbances in people living with dementia: A systematic review and meta-analysis. Geriatric Nursing, 51, 76-83.<br>2. Whelan, S., Murphy, K., Barrett, E., Krusche, C., Santorelli, A., & Casey, D. (2018). Factors affecting the acceptability of social robots by older adults including people with dementia or cognitive impairment: a literature review. International Journal of Social Robotics, 10(5), 643-668.<br>3. Vandemeulebroucke, T., Dzi, K., & Gastmans, C. (2021). Older adults' experiences with and perceptions of the use of socially assistive robots in aged care: A systematic review of quantitative evidence. Archives of Gerontology and Geriatrics, 95, 104399.

	4. Xie, B., Tao, C., Li, J., Hilsabeck, R. C., & Aguirre, A. (2020). Artificial intelligence for caregivers of persons with Alzheimer's disease and related dementias: systematic literature review. JMIR medical informatics, 8(8), e18189.
Substantial overlap with other included reviews (duplicate evidence)	1. Gochoo, M., Vogan, A. A., Khalid, S., & Alnajjar, F. (2020, September). AI and robotics-based cognitive training for elderly: a systematic review. In 2020 IEEE/ITU International Conference on Artificial Intelligence for Good (AI4G) (pp. 129-134). IEEE.<br>(duplicate evidence with Vogan et al., 2020)
Not retrieved, full text not available	1. Chow, K. K., Ip, C. S., Yau, C. T., Zeng, J., & Zhong, J. (2025). A Systematic Review of Using Human-Robot Interaction for Cognitive Training for Elderly with Mild Cognitive Impairment. In International Conference on Intelligent Robotics and Applications (pp. 30-41). Springer, Singapore.<br>2. Park, K., & Lee, S. (2019). Effect of socially assistive robot intervention on people with dementia: a meta-analysis. Alzheimers Dementia, 15(7), P574.

Supplementary Table S3. AMSTAR-2 results for individual studies included in the umbrella review

**Review**	1	2*	3	4*	5	6	7*	8	9*	10	11*	12	13*	14	15*	16	Overall confidence
**Moyle et al. (2017)**	N	Y	Y	Y	Y	N	N	Y	N	N	N/A	N/A	Y	Y	N/A	Y	
**Kang at al. (2019)**	Y	N	Y	Y	N	Y	N	Y	Y	N	N/A	N/A	Y	Y	N/A	Y	**Critically low**
**Leng et al. (2019)**	Y	N	Y	Y	Y	Y	N	Y	Y	N	Y	N	Y	Y	N	Y	**Critically low**

**Pu et al. (2019)**	Y	Y	Y	Y	Y	Y	N	Y	Y	N	Y	N	Y	Y	N/A	Y	**Low**
**Vogan et al. (2020)**	N	N	Y	N	N	N	N	Y	N	N	N/A	N/A	Y	Y	N/A	N	**Critically low**
**Ghafurian et al. (2021)**	N	N	Y	Y	N	N	N	Y	N	N	N/A	N/A	N	Y	N/A	Y	**Critically low**
**Lu et al., 2021**	Y	N	Y	Y	Y	Y	N	Y	Y	N	Y	N	Y	Y	Y	Y	**Critically low**
**Ong et al. (2021)**	Y	N	Y	Y	Y	Y	N	Y	Y	N	Y	N	Y	Y	N/A	Y	**Critically low**
**Saragih et al. (2021)**	Y	Y	Y	Y	Y	Y	N	Y	Y	N	Y	N	Y	Y	Y	Y	**Low**
**Lee et al. (2022)**	Y	N	Y	N	Y	N	N	Y	Y	N	Y	N	P/Y	Y	Y	Y	**Critically low**
**Yu et al. (2022)**	Y	Y	Y	Y	Y	Y	N	Y	Y	N	Y	P/Y	Y	Y	N	Y	**Critically low**
**Figliano et al. (2023)**	N	N	Y	Y	Y	N	N	Y	Y	N	N/A	N/A	Y	Y	N/A	Y	**Critically low**

**Hsieh et al. (2023)**	Y	Y	Y	Y	Y	N	N	Y	Y	Y	N	N	Y	Y	N/A	Y	**Critically low**
**Noh & Shim (2023)**	Y	N	Y	Y	Y	Y	N	Y	Y	N	Y	N	Y	Y	N	Y	**Critically low**
**Yen et al. (2024)**	Y	N	Y	Y	Y	Y	N	Y	Y	N	Y	P/Y	Y	Y	Y	Y	**Critically low**
**Fan et al. (2025)**	Y	Y	Y	Y	Y	Y	N	Y	Y	N	Y	N	Y	Y	Y	Y	**Low**

The definition of each item is as follows:

1. Did the research questions and inclusion criteria for the review include the components of PICO?
2*. Did the report of the review contain an explicit statement that the review methods were established prior to the conduct of the review and did the report justify any significant deviations from the protocol?
3. Did the review authors explain their selection of the study designs for inclusion in the review?
4*. Did the review authors use a comprehensive literature search strategy?
5. Did the review authors perform study selection in duplicate?
6. Did the review authors perform data extraction in duplicate?
7*. Did the review authors provide a list of excluded studies and justify the exclusions?
8. Did the review authors describe the included studies in adequate detail?
9*. Did the review authors use a satisfactory technique for assessing the risk of bias (RoB) in individual studies that were included in the review?
10. Did the review authors report on the sources of funding for the studies included in the review?
11*. If meta-analysis was performed did the review authors use appropriate methods for statistical combination of results?
12. If meta-analysis was performed, did the review authors assess the potential impact of RoB in individual studies on the results of the meta-analysis or other evidence synthesis?
13*. Did the review authors account for RoB in individual studies when interpreting/ discussing the results of the review?

14. Did the review authors provide a satisfactory explanation for, and discussion of, any heterogeneity observed in the results of the review?
15*. If they performed quantitative synthesis did the review authors carry out an adequate investigation of publication bias small study bias and discuss its likely impact on the results of the review?
16. Did the review authors report any potential sources of conflict of interest, including any funding they received for conducting the review?
***Critical domain**
The methodological quality evaluation criteria are as follows:
High—No or one non-critical weakness: the systematic review provides an accurate and comprehensive summary of the results of the available studies that address the question of interest;
Moderate—More than one non-critical weakness: the systematic review has more than one weakness but no critical flaws. It may provide an accurate summary of the results of the available studies that were included in the review;
Low—One critical flaw with or without non-critical weaknesses: the review has a critical flaw and may not provide an accurate and comprehensive summary of the available studies that address the question of interest;
Critically low—More than one critical flaw with or without non-critical weaknesses: the review has more than one critical flaw and should not be relied on to provide an accurate and comprehensive summary of the available studies.

Supplementary Table S4. ROBIS results for individual studies included in the umbrella review

		Phase 2			Phase 3
**Review**	**Eligibility Criteria**	**Identification & Selection**	**Data Collection & Appraisal**	**Synthesis & Findings**	**Overall Risk of Bias**
**Moyle et al. (2017)**	Low	Low	High	Low	**High**
**Kang et al. (2019)**	Low	High	Low	Low	**High**
**Leng et al. (2019)**	Low	Low	Low	High	**High**
**Pu et al. (2019)**	Low	High	Low	High	**High**

**Vogan et al. (2020)**	High	High	High	High	**High**
**Ghafurian et al. (2021)**	Low	High	High	Low	**High**
**Lu et al. (2021)**	Low	Low	Low	High	**High**
**Ong et al. (2021)**	Low	Low	Low	High	**High**
**Saragih et al. (2021)**	Low	Low	Low	High	**High**
**Lee et al. (2022)**	Low	High	High	High	**High**
**Yu et al. (2022)**	Low	Low	Low	High	**High**
**Figliano et al. (2023)**	Low	Low	Low	Low	**Low**
**Hsieh et al. (2023)**	Low	Low	High	High	**High**
**Noh & Shim (2023)**	Low	Low	Low	High	**High**
**Yen et al. (2024)**	Low	Low	Low	Low	**Low**
**Fan et al. (2025)**	Low	Low	Low	Low	**Low**

Supplementary Table S5. Citation matrix of all primary studies (rows) included for each review (columns) used to calculate the CCA

		Moyle 2017	Kang et al. (2019)	Leng et al. (2019)	Pu et al. (2019)	Vogan et al. (2020)	Ghafurian et al. (2021)	Lu et al. (2021)	Ong et al. (2021)	Saragih et al. (2021)	Lee et al. (2022)	Yu et al. (2022)	Figliano et al. (2023)	Hsieh et al. (2023)	Noh & Shim (2023)	Yen et al. (2024)	Fan et al. (2025)
1	Wada et al. (2002)						**1**										
2	Tamura et al. (2004)											**1**					
3	Wada et al. (2005)						**1**										
4	Marti et al. (2006)						**1**										
5	Odetti et al. (2007)						**1**						**1**				
6	Tomita et al. (2007)										**1**						
7	Wada & Shibata (2007)						**1**										
8	Banks et al. (2008)				**1**											**1**	
9	Schermerhorn et al. (2008)					**1**											
10	Kramer et al. (2009)											**1**					
11	Song et al. (2009)											**1**					
12	Tapus (2009)						**1**										
13	Chan & Nejat (2010)						**1**										
14	Marx et al. (2010)											**1**					

15	Chan & Nejat (2011)						**1**										
16	Chan et al. (2011)						**1**										
17	Bemelmans et al. (2012)											**1**					
18	Chan & Nejat (2012)						**1**										
19	de Sant'Anna et al. (2012)											**1**					
20	Gross et al. (2012)	**1**															
21	Inoue et al. (2012)											**1**					
22	Khosla et al. (2012)						**1**										
23	McColl et al. (2012)						**1**										
24	Shibata (2012)						**1**										
25	Takenobu et al. (2012)											**1**					
26	Tanaka et al. (2012)				**1**	**1**					**1**						
27	Begum et al. (2013)						**1**										
28	Bemelmans et al. (2013)											**1**					
29	Chang et al. (2013)						**1**										
30	Martin et al. (2013)						**1**										

31	McColl & Nejat (2013)						**1**										
32	McColl et al. (2013)						**1**										
33	Moyle et al. (2013)		**1**	**1**	**1**			**1**	**1**	**1**		**1**		**1**			
34	Šabanović et al. (2013)					**1**	**1**						**1**				
35	Schroeter et al. (2013)	**1**															
36	Robinson et al. (2013)	**1**			**1**			**1**	**1**			**1**		**1**		**1**	
37	Broadbent et al. (2014)				**1**												
38	Chang et al. (2014)						**1**										
39	Khosla et al. (2014)						**1**										
40	Louie et al. (2014)						**1**										
41	Moyle et al. (2014)	**1**					**1**					**1**					
42	Sung et al. (2014)					**1**											

43	Takayanagi et al. (2014)					**1**	**1**					**1**	**1**				
44	Begum et al. (2015)						**1**					**1**					
45	Bemelmans et al. (2015)						**1**	**1**	**1**			**1**					
46	Chang & Šabanovic (2015)						**1**										
47	Gustafsson et al. (2015)											**1**					
48	Jøranson et al. (2015)		**1**	**1**	**1**		**1**	**1**	**1**	**1**				**1**	**1**	**1**	**1**
49	Kim et al. (2015)					**1**							**1**				
50	Naganuma et al. (2015)						**1**										
51	Oh et al. (2015)										**1**						
52	Pino et al. (2015)						**1**										
53	Rudzicz et al. (2015)											**1**					
54	Valentí Soler et al. (2015)		**1**	**1**	**1**	**1**	**1**	**1**	**1**	**1**	**1**	**1**	**1**	**1**	**1**		**1**

55	Bemelmans et al. (2016)											**1**					
56	Chu et al. (2016)											**1**					
57	Hamada et al. (2016)						**1**										
58	Hebesberger et al. (2016)						**1**										
59	Jøranson et al. (2016a)											**1**					
60	Jøranson et al. (2016b)		**1**				**1**	**1**	**1**	**1**		**1**		**1**			**1**
61	Lane et al. (2016)						**1**					**1**					
62	Libin & Cohen-Mansfield (2016)											**1**					
63	Moyle et al. (2016)											**1**					
64	Peterson et al. (2016)					**1**											
65	Thodberg et al. (2016a)							**1**						**1**			
66	Thodberg et al. (2016b)				**1**			**1**	**1**					**1**			
67	Abdollahi et al. (2017)						**1**						**1**				

68	Chu et al. (2017)						**1**						**1**				
69	Khosla et al. (2017)											**1**	**1**				
70	Kouroupetroglou et al. (2017)											**1**					
71	Liang et al. (2017)		**1**	**1**	**1**	**1**	**1**	**1**	**1**	**1**		**1**		**1**	**1**		**1**
72	Moyle et al. (2017)		**1**	**1**	**1**		**1**	**1**	**1**	**1**		**1**		**1**	**1**	**1**	**1**
73	Perugia et al. (2017a)						**1**										
74	Perugia et al. (2017b)						**1**										
75	Petersen et al. (2017)		**1**	**1**	**1**		**1**	**1**	**1**	**1**				**1**	**1**	**1**	**1**
76	Rouaix et al. (2017)						**1**										
77	Wang et al. (2017)						**1**					**1**					
78	De Kok et al. (2018)						**1**						**1**				
79	Demange et al. (2018)											**1**					
80	In Soon et al. (2018)											**1**					
81	Jones et al. (2018)							**1**	**1**			**1**		**1**			
82	Koh and Kang (2018)								**1**		**1**	**1**					
83	Mervin et al. (2018)								**1**	**1**							**1**
84	Moyle et al. (2018)		**1**				**1**	**1**	**1**	**1**		**1**		**1**			**1**

85	Paletta et al. (2018)						**1**										
86	Polak et al. (2018)					**1**											
87	Tanigaki et al. (2018)												**1**				
88	Antona et al. (2019)						**1**										
89	Demange et al. (2019)											**1**					
90	D'Onofrio et al. (2019)						**1**					**1**					
91	Hung et al. (2019)											**1**					
92	Kase et al. (2019)								**1**								
93	Khosla et al. (2019)											**1**					
94	Law et al. (2019)											**1**					
95	Mannion et al. (2019)						**1**										
96	Moyle et al. (2019)						**1**		**1**			**1**		**1**			
97	Pino et al (2019)					**1**											
98	Pu et al. (2019)											**1**					
99	Arthanat et al. (2020)											**1**					
100	Chen et al. (2020)									**1**		**1**		**1**	**1**	**1**	**1**

101	Barakova et al. (2020)											**1**					
102	Casey et al. (2020)											**1**					
103	Jøranson et al., (2020)									**1**							
104	Ke et al. (2020)									**1**		**1**	**1**				**1**
105	Lee et al. (2020)									**1**			**1**		**1**		
106	Moyle et al. (2020)											**1**					
107	Obayashi et al. (2020)								**1**								
108	Rico et al. (2020)											**1**					
109	Palestra et al. (2020)												**1**				
110	Pino et al. (2020)												**1**				
111	Pou-Prom et al. (2020)											**1**					
112	Pu et al. (2020)								**1**	**1**		**1**			**1**	**1**	**1**

113	Tummers et al. (2020)											**1**					
114	Feng et al (2021)											**1**					
115	Fogelson et al. (2021)											**1**					
116	Hammarlund et al. (2021)											**1**					
117	Inoue et al. (2021)											**1**					
118	Jøranson et al. (2021)																**1**
119	Kelly et al. (2021)											**1**					
120	Khosla et al. (2021)						**1**										
121	Manca et al. (2021)												**1**				
122	Nakamura et al. (2021)											**1**					
123	Natarajan et al. (2021)											**1**					
124	Otake-Matsuura et al (2021)										**1**						

125	Papadopoulos et al (2021)															**1**	
126	Park et al (2021)										**1**		**1**		**1**		
12	Pu et al. (2021)									**1**							**1**
128	Schramek et al. (2021)											**1**					
129	Schuurmans et al. (2021)											**1**					
130	Tulsulkar et al (2021)												**1**				
131	Van Assche et al. (2021)												**1**				
132	Yoshii et al (2021)										**1**						
133	Zuschnegg et al. (2021)											**1**					
134	Bradwell et al. (2022)																**1**
135	Lin et al. (2022)												**1**				
136	Pu et al. (2022)											**1**					

137	Sugiyama and Nakamura (2022)																**1**
